# Horse-related injury patterns: a single center report

**DOI:** 10.1186/s13018-023-03549-3

**Published:** 2023-02-02

**Authors:** M. F. Hoffmann, M. Bernstorff, N. Kreitz, B. Roetman, T. A. Schildhauer, K. E. Wenning

**Affiliations:** 1 Berufsgenossenschaftliches Universitätsklinikum Bergmannsheil Bochum, Bürkle-de-La-Camp-Platz 1, 44789 Bochum, Germany; 2 Schön Klinik Eilbek, Denhaide 120, 22081 Hamburg, Germany; 3 Mathias Spital Rheine, Frankenburgstraße 31, 48431 Rheine, Germany

**Keywords:** Horse-related, Injury, Rider, Sports, Trauma, Equestrian, Spinal injury, Amputation

## Abstract

**Background:**

For ages, humankind and horses have been closely related to occupational and recreational activities. The dangers of engaging with horses have been previously reported. Among sporting activities, horse riding is well-known for its risks. Despite multiple recommendations to wear protective gear, horse-related activities still comprise the risk of severe injuries. This study aimed to examine: (1) if specific mechanisms are correlated to particular injury patterns and (2) if injury types are related to patient demographics.

**Methods:**

From one level I trauma center, between July 2019 and July 2022 (3 years) all emergency reports and discharge letters were retrospectively reviewed by full-text search regarding horse-related injuries. Patient demographics, body mass index, trauma mechanism, injury types, and initiated treatment were extracted from medical records and analyzed.

**Results:**

During the study period, 95 patients with 99 horse-related injuries were included. The overwhelming majority of the patients was female (93.7%). Age averaged 35.3 years (range 6 to 71). BMI was 23.6 kg/m^2^. Inpatient treatment was required in 60.6%. Length of hospital stay averaged 10 days. Surgical treatment was performed in 55 patients (55.6%). Open reduction and internal fixation was the most common procedure (74.5%). Trauma mechanism was fall from a horse followed by being hit by a horse (60.6% and 23.2%, respectively). Injured upper extremities counted up for 52.5% followed by spinal and pelvic injuries (23.2%). Spinal and pelvic injuries were related to fall from a horse (*p* < 0.001). Injuries to the lower extremities were predominantly caused by a kick of the horse when the rider was unmounted (*p* = 0.001) and negatively related to a fall from a horse (*p* = 0.002). Ten patients got their fingers tangled while holding the reins and suffered from injuries to the upper extremity (*p* < 0.001). Three of them required an amputation (30%).

**Conclusion:**

Despite the fact that patients are young and healthy, horse related injuries must not be underestimated. In our study, almost two-thirds of the patients required inpatient treatment and 50% underwent surgery. We could show that patient age was related to injury severity according to the Abbreviated Injury Scale (AIS). Spinal and pelvic injuries were significantly related to a fall from a horse with a significantly greater trauma impact according to the AIS. Therefore, these severe entities need to be ruled out in such events. Accidents caused by holding the reins, may result in serious injuries to the hand with 30% requiring an amputation. Doctors need to be aware of possible horse-related injury patterns to reduce morbidity.

## Background

For ages, mankind has been domesticating horses for occupational and recreational activities. During the recent decades, in industrial countries the relationship with horses has focused on sports and leisure. The dangers of engaging with horses and their unpredictable behavior when it comes to unforeseen circumstances have been previously reported [1, 2]. Among sporting activities, horse riding is well known for its risks in regards to their height, velocity, kicking force, and weight [2, 3]. Despite multiple recommendations to wear personal protective equipment, horse-related activities still comprise the risk of severe injuries comparable to motorized sports [4–6].

While the awareness of risk factors associated with horse-related injuries may be useful for prevention among horseback riders [7], the knowledge of risk factors among medical staff may help with diagnostics and may reduce morbidity. This study aimed to examine: (1) if specific mechanisms are correlated to particular injury patterns and (2) if injury types are related to different patient demographics.

## Methods

This study was an Institutional Review Board approved retrospective cohort study of consecutive patients undergoing treatment for horse-related injuries in one level I and referral trauma center in an urban area. During the study period, from the start of July 2019 through to the end of June 2022 (3 years) a total of 102 injuries were identified by full-text search of patient medical records (MEDICO, CGM Europe) that had treatment for horse-related injuries. Inclusion criteria were outpatient or inpatient treatment for injuries caused by horse-riding or during associated nonriding activities (e.g. feeding, grooming, shoeing, etc.). Exclusion criteria were injuries to persons not involved with the normal handling of the animals or injuries that occurred fatefully. No patients were phoned in specifically for this study; all data were obtained from preexisting medical records and radiographs.

### Clinical data assessment

Patient demographics, injury mechanism and pattern, and medical treatment were extracted from the hospital documentation program (MEDICO, CGM Europe). Gender, age, Body-Mass-Index (BMI), insurance status, and injury mechanism were collected as descriptive data. Injury pattern, inpatient treatment, length of hospital stay, and surgeries performed were utilized as outcomes data. Radiographs were reviewed utilizing the picture archiving and communication system (PACS) (IMPAX 6.6.1, AGFA HealthCare N.V., Belgium).

### Statistical analysis

For statistical analysis, Excel (Microsoft® Office Excel©, Version 16.44) and SPSS version 27.0 (IBM, Chicago, IL) were utilized. To compare continuous variables (age, BMI, inpatient stay) T-test was performed. For nominally and ordinally scaled data, the *C*ℎ*i*^*2*^ test or *Fischer's exact test* was applied. A significance threshold of *p* < 0.05 was selected for all statistical tests and the *p* values determined were rounded to three decimal places.

## Results

**Fig. 1:**
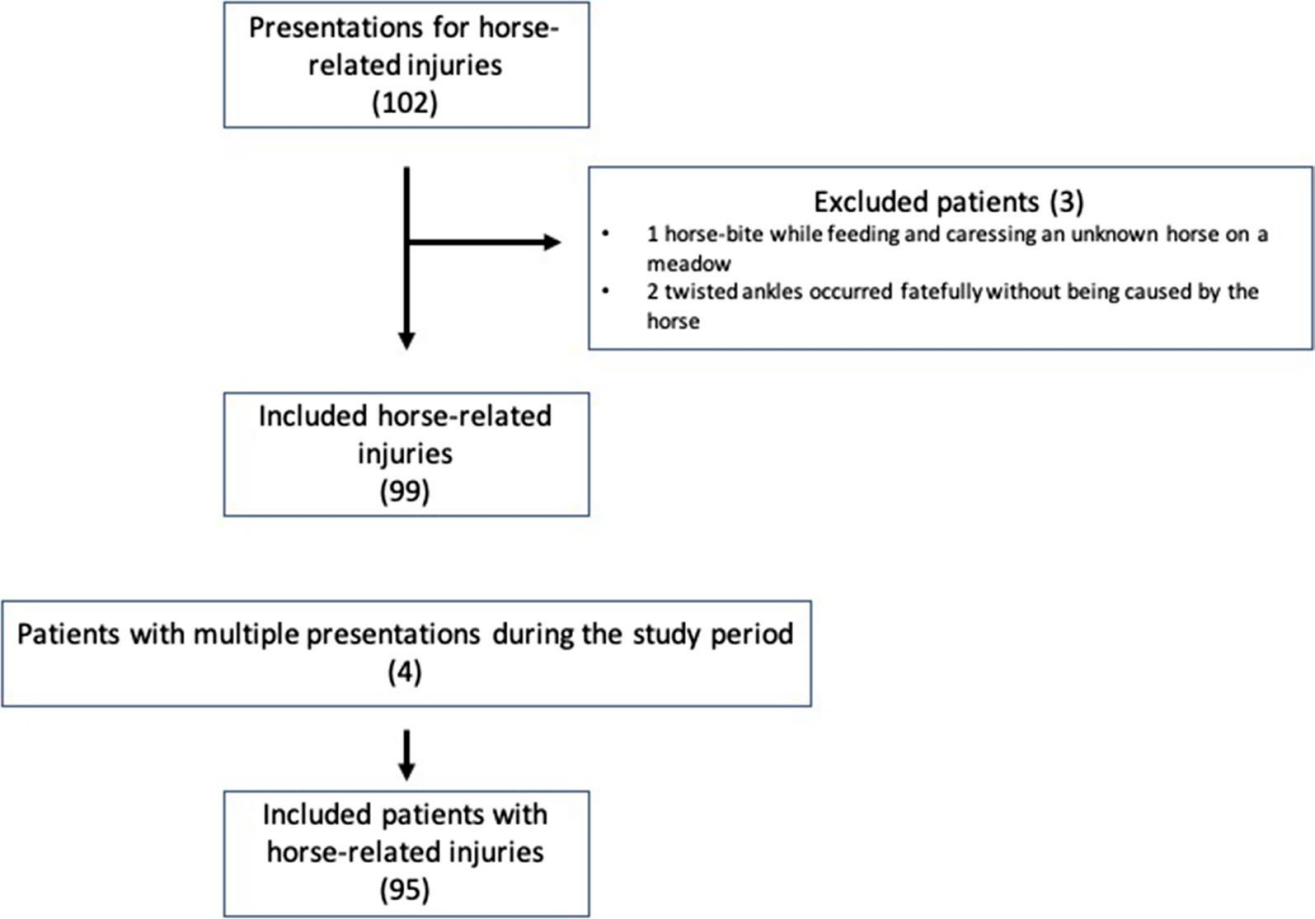
Flow diagram showing numbers of included patients and injuries

During the study period, full-text search revealed 102 presentations for horse-related injuries. Three patients were excluded. One patient suffered from a horse-bite while feeding and caressing an unknown horse on a meadow. Two patients twisted their ankles while walking next to their horse or longeing a horse. The latter injuries occurred fatefully without being caused by the horse. (Fig. 1). Four patients presented twice during the study period for different accidents, which left us with 99 horse-related injuries in 95 patients. The overwhelming majority of the patients was female (93.7%). Age averaged 35.5 years (range 6–71) with 20 patients younger than 18 years (21.1%). BMI was 23.6 kg/m^2^ (SD ± 4.03). One third of the patients (32/95) had a private health insurance, which is three times as much as in the normal population (10.5%) [8].

### Trauma mechanism

Trauma mechanism was falling from a horse followed by being kicked or trampled by a horse (60.6% and 23.2%, respectively). Ten patients got their fingers tangled while holding the reins. Other trauma mechanisms were observed in 6 patients including getting a foot trapped in a stirrup, being hit by the head of the horse, and being squashed while being in a horse stall. Injured upper extremities counted up for 52.5% followed by spinal and pelvic injuries (23.2%). Spinal and pelvic injuries were related to fall from a horse (*p* < 0.001) and negatively related to injuries of the lower extremities (*p* = 0.002). Injuries to the lower extremities were predominantly caused by a kick from the horse when the rider was unmounted (*p* = 0.001) (Table 1).

**Table 1:** Correlation of trauma mechanism and injured body region (fall from horse is negatively correlated to injuries of the lower extremity and kick by a horse was negatively related to spinal or pelvic injury)

Injured body region		Trauma mechanism		
		Fall from horse	Kick by horse	Rein injury
		60 (60.6%)	23 (23.2%)	10 (10.1%)
Upper extremity	52 (52.5%)	31 (51.7%) *p* = 0.844	9 (39.1%) *p* = 0.160	10 (100.0%) *p* = 0.001
Lower extremity	20 (20.2%)	6 (10.0%) *p* = 0.002	11 (47.8%) *p* = 0.001	0 (0.0%) *p* = 0.116
Spine/pelvis	23 (23.2%)	22 (36.7%) *p* < 0.001	1 (4.3%) *p* = 0.021	0 (0.0%) *p* = 0.111
Head	9 (9.1%)	6 (10.0%) *p* = 0.735	2 (8.7%) *p* = 0.967	0 (0.0%) *p* = 0.593

Injury severity varied from 1 to 5 according to the Abbreviated Injury Scale (AIS) with an average of 2.13 (SD ± 0.95) and was related to the age of the patients (*p* = 0.015). AIS was not related to BMI (*p* = 0.595). Sixty patients were admitted for inpatient treatment (60.6%). Patients treated in the hospital had a higher AIS compared to those treated as outpatients (AIS 2.45 vs. 1.64, respectively; *p* < 0.001). Surgical treatment was performed in 55 patients (55.6%). Open reduction and internal fixation (ORIF) was the most common procedure (74.5%) (Table 2). Length of hospital stay averaged 9.9 days (SD ± 26.7).

**Table 2:** Surgical procedures performed for horse-related injuries

	Performed surgical procedures	Percentage
Surgery (total)	55	55.6% of 99 injuries
Open reduction and internal fixation	41	74.5%
External fixator	2	4.8%
Closed reduction and internal fixation	3	7.1%
Arthroscopy	3	7.1%
Finger amputation	3	7.1%
Debridement/vacuum	1	2.4%
Arterial ligature for extensive bleeding	1	2.4%
External ventricular drainage	1	2.4%

### Injury types

Patients with *spinal or pelvic injuries* had a higher AIS (2.48 vs. 2.03, respectively; *p* = 0.046) and were treated for significantly longer in hospital (25 days) compared to other injuries (*p* = 0.019). This was caused by two patients with complete and incomplete tetraplegia after trauma to the cervical spine. Patients with spinal or pelvic injuries tended to be older (38.5 vs 34.4 years) but that was not significant (*p* = 0.280). Eleven patients with injuries of the spine and pelvis underwent surgery (47.8%) (Table 3).

**Table 3:** Surgical procedures in patients with injuries of the spine and pelvis

Number	Sex	Age	Injury	Procedure
1	Female	16	L-1 fracture	Posterior stabilization T-12 to L-2/3
2	Female	58	Complete tetraplegia, C2, T 3–6 and T-9 fracture	Posterior stabilization C0–C3, posterior stabilization T-4 to T-7
3	Female	43	L-2 fracture	Posterior stabilization L-1 to L-3
4	Female	56	L-3 and L-4 fracture	Posterior stabilization L-3/4
5	Female	55	Fractures of the right transverse process T-3 to 8, bilateral pelvic fracture, (lateral clavicle fracture)	Right SI-screw, ORIF right clavicle
6	Female	55	Incomplete tetraplegia, rupture of the intervertebral disc C3/4 and C-5/6	Decompression, posterior stabilization, and fusion C-3 to C-7
7	Male	57	Open book injury with vertical instability	ORIF of the symphysis, SI-screw
8	Female	17	Fracture T-5/6	Posterior stabilization T4 to T-7
9	Female	16	Fractures of T-8, L-1, L-2	Posterior stabilization T12 to L-3
10	Female	50	Instability C-5/6, (skull-base fracture)	Anterior fusion C-5/6
11	Female	55	Complex right pelvic injury	Lumbopelvic fixation

The majority of patients (52, 52.5%; respectively) presented for *injuries to the upper limb*. Thereof, thirty-five patients were admitted for inpatient treatment (67.3%). Length of hospital stay was shortest for patients with upper extremity injuries and averaged 4.1 days (*p* = 0.041). Surgery was performed in 34 patients (65.4%) (Table 4). Injuries involving the hand required surgery most frequently (35.3%). This results also from rein injuries. Ten patients got their fingers tangled while holding the reins and therefore suffered from injuries to the upper extremity (*p* = 0.001). Three of them required an amputation (30%).

**Table 4:** Anatomic regions of surgical procedures performed for injuries to the upper extremity

Upper extremity (52)	Performed surgical procedures	Percentage (%)
Surgery (total)	34	65.4
Shoulder girdle	8	23.5
Humerus	3	8.8
Elbow	6	17.6
Forearm	6	17.6
Hand	12	35.3

*Lower extremity injuries* were related to a kick from a horse or being trodden on a foot (*p* = 0.001) and occurred in 20 patients (20.2%). Nine patients (45%) were admitted to the hospital for subsequent surgery. Open reduction and internal fixation of the ankle joint was performed in two patients. Three patients underwent ORIF for tibial shaft and tibial plateau fractures. Additionally, one patient required an anterior cruciate ligament replacement after being knocked down while walking her horse. Two patients suffered from foot injuries and one patient underwent a hip arthroscopy for a flake fracture and microfracturing of the femoral head.

Because of *head injuries*, nine patients (9.1%) were presented in the emergency department. Mean age of patients with head trauma was 41.7 years compared to 34.7 years for the rest of the study population (*p* = 0.304). Head trauma had the lowest AIS (1.89) and varied from contusion (5 patients) and head wound with active bleeding (one patient) to severe traumatic brain injury with intracranial hemorrhage. One patient (1%) deceased due to traumatic brain injury with brainstem lesion. Therefore, the mortality rate of our study was 1.0%.

## Discussion

Despite the fact that injuries related to equestrian accidents form only a small percentage of attendances to the emergency department especially in urban areas, injury patterns should not be underestimated and consequences of these injuries can be profound [6, 9]. Most of the horse-related injuries (60.6%) occurred while mounted on horseback, followed by being kicked by a horse. This is in concordance with a US National Electronic Injury Surveillance System (NEISS) data analysis and previous studies [4, 9]. This single center experience reveals that injury patterns vary between mounted and unmounted individuals. While falling from a horse leads to more severe spinal and pelvic injuries, tangling up in reins resulted in upper extremity injuries (1). No gender specific differences in injury types were observed, but injury severity was related to patient age according to the AIS (2).

Regarding the female majority in our study population, differences of horse-related activities exist between nations and countries. Horseback riding seems to be a male sport in Israel with 79% injured boys [10]. Whereas, a 10-year national trauma database analysis in the US showed a 50/50 distribution between males and females [1]. Studies from Australia found 54–59% females with horse-related injuries [7, 11]. In contrast hereto, there is a predominance of females in Europe. Two studies from The Netherlands and France found 81% females with equestrian-related injuries [2, 12]. Whereas, a Swedish study revealed up to 98% injured females [13]. In Germany, horseback riding is a popular sport especially for younger females (80%) [14]. Therefore, our study population of 93% females is reasonable but makes it almost impossible to find any gender specific differences.

Despite, the worldwide trend of people becoming more overweight and obese, the observed BMI in our study averaged 23.6 kg/m^2^. This is less than the average BMI of 26.01 kg/m^2^ for women in Germany [15], which might be related to the fact that horseback riding in Germany is expensive and an upper-class sport. The high rate of privately insured patients supports this theory. This may also be accentuated by the younger age (35.3 years) of the study population. The younger age of patients with horse-related injuries is a previously reported characteristic [9, 11, 16]. Additionally, in contrast to previous findings, that increasing BMI was associated with an increase in injury severity [17], in our study the AIS was not related to BMI.

Most studies report head trauma as the most common injury [1, 9, 10, 18]. Especially studies with its focus on children reported up to 30% head injuries [10, 16, 18]. In contrast hereto, only 10% of the patients in our study presented with head injuries, but our study combined children and adults. Additionally, Moss et al. reported already in 2002 a significant reduction of head injuries related to the development of improved protective helmets [9]. We could show that head injuries had the lowest AIS compared to other injuries. Still, traumatic brain injury remains the leading external cause of death [3, 19] and Lin et al. summed up, that in horseback riders traumatic brain injuries remain the most significant cause of mortality [20]. In line with that, in the current study one patient deceased due to traumatic brain injury with brainstem lesion. The resulting mortality rate (1%) of our study also reflects the mortality rate of equestrian related injuries of about 1% in previous studies [2, 7].

**Fig. 2:**
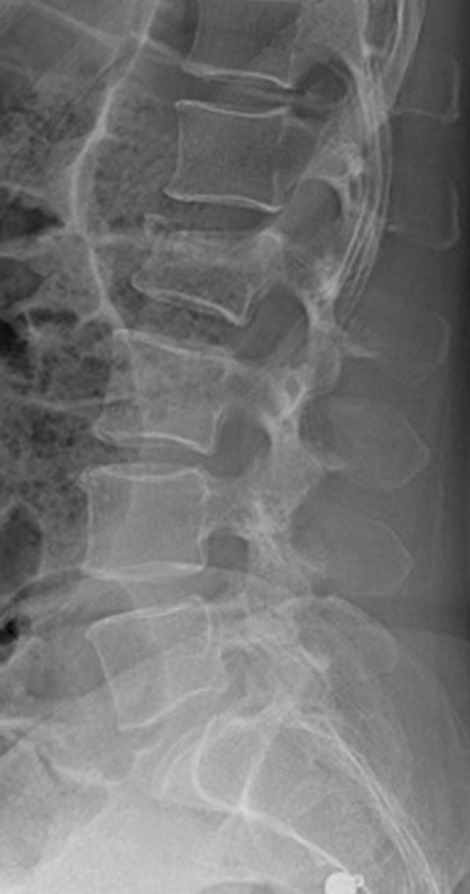
Incomplete compression fracture (AO Spine Type A3) of L2 after fall from a horse

Falling from a horse was significantly related to spinal or pelvic injuries. Regarding spinal injuries previous studies reported more lumbar and thoracic than cervical spine injuries with a predominance of the thoracolumbar junction [6, 21] (Fig. 2). The average hospital admittance time (25 days) for spinal and pelvic injuries in our study was significantly longer than for other body regions but comparable to other European studies (11–43 days) [6]. The extended length of stay was caused by the significantly greater AIS and was accentuated by two patients with complete and incomplete tetraplegia after trauma to the cervical spine, who started early spinal rehabilitation. Previous studies also found 3% severely neurologically impaired patients [1]. This supports the conclusion by Lin et al., that in horseback riders spinal cord injuries are a significant cause of long-term morbidity [20].

**Fig. 3:**
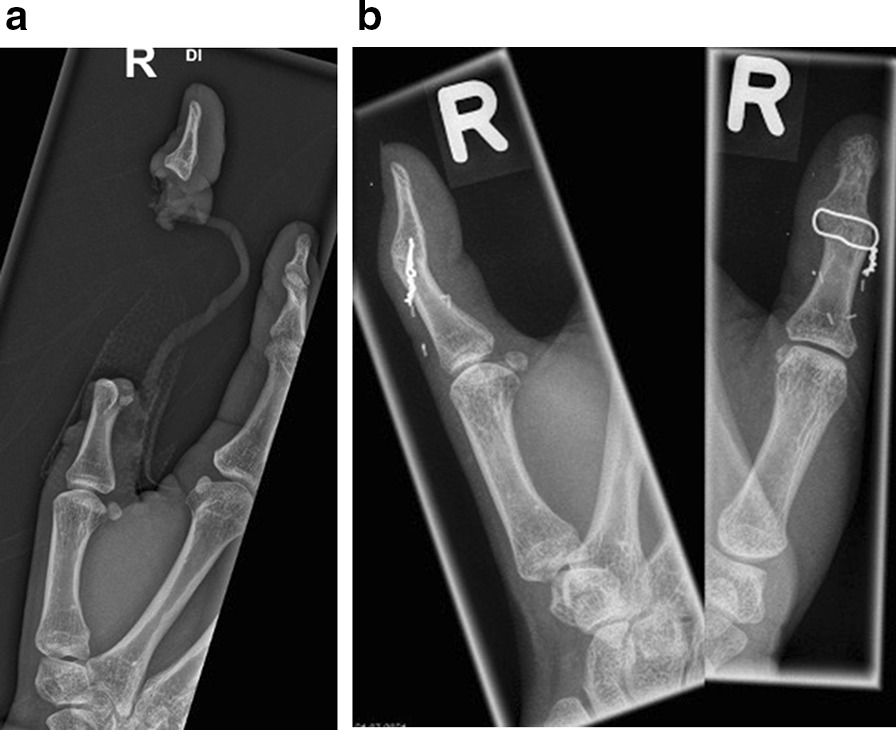
**a** Subtotal amputation of the thumb after entanglement with reins. **b** Salvage of the thumb with arthrodesis of the distal joint

In our study, injuries to the upper limb were the most common injuries. This is in concordance with other European studies from France, Austria, and Hungary [12, 22] and might reflect a difference in personal protective equipment. Moss et al. already described a decline in head injuries and an increase in the number and severity of upper limb injuries [9]. To our knowledge, no data exist comparing differences between nations or regions regarding the utilization of helmets. In a study by Kiss et al. Children in Austria wore a helmet in 70% compared to 43% in Hungary [22]. Abu-Zidan and Rao [7] reported a helmet use of 73% in an Australian study. Injuries to the upper extremity seemed to be less severe. Therefore, length of hospital stay was shortest for patients with upper extremity injuries. Surgery was performed in 34 patients (65.4%). Injuries involving the hand required surgery most frequently (35.3%). While many types of injuries are common for horseback-riding and grooming-related activities, literature regarding injuries to the fingers and thumb is sparse. Mangal et al. [23] reported one case with traumatic multi-finger amputations after trying to rein a horse. Abu-Zidan and Rao [7] reported seven patients (3%) that were entangled with reins and needed hospitalization, but did not report any injuries or treatment. The current study reveals that 10% of the injuries were caused by fingers tangled with reins or ropes (Fig. 3a, b). Most of them resulted in injuries to the ligaments and tendons, which could be reinserted in 40% of the cases. Still, three of them required an amputation (30%). The injury mechanism at it may be comparable to rodeo roping thumb injuries [24].

Lower limb injuries occurred in 20% of the patients. Altgärde et al. [13] reported 24% in adults and 19% in children. A US database query revealed 16.4% injured lower extremities [16]. Lower limb injuries were related to a kick from a horse or being trodden on a foot. To our knowledge, this has not been described before. This is supported by Lang et al. [11], who found a significantly greater number of lower extremity injuries in adults compared to children. This may be related to the fact, that adults are more involved in grooming and feeding.

We acknowledge the limitations of our study. The major limitation of this study was its retrospective design. While in the study by Lang et al. approximately 20% of the injuries occurred while working, the presented study was performed in an urban setting, where horseback riding is a popular recreational activity. Therefore, we are not able to report injury patterns for occupational activities. Additionally, due to the fact that our trauma center includes orthopedics, hand surgery, plastic surgery, and neurosurgery but no ENT department, isolated facial injuries treated by an ENT-specialist might be missed. Therefore, further data and studies are warranted.

Doctors should counsel patients involved in horseback riding to become thoroughly aware of the risks of equestrian-related sport and to understand the importance of appropriate training and safety gear [3].

## Conclusion

Despite the fact that patients are young and healthy, horse related injuries must not be underestimated. In our study, almost two-thirds of the patients required inpatient treatment and 50% underwent surgery. We could show that patient age was related to injury severity according to the Abbreviated Injury Scale (AIS). Spinal and pelvic injuries were significantly related to a fall from a horse with a significantly greater trauma impact according to the AIS. Therefore, these severe entities need to be ruled out in such events. Accidents caused by holding the reins, may result in serious injuries to the hand with 30% requiring an amputation. Doctors need to be aware of possible horse-related injury patterns to reduce morbidity.

## Data Availability

All data are available within the manuscript.
